# Relationship between digestive diseases and COVID-19 severity and mortality: A protocol for systematic review and meta-analysis

**DOI:** 10.1097/MD.0000000000023353

**Published:** 2020-11-25

**Authors:** Jinjuan Li, Jia Yue, Shunan Zhang, Jianjun Wu, Rongna Lian, Ruinian Zhang, Peng Cheng

**Affiliations:** a School of Public Health, Gansu University of Chinese Medicine; b The First Clinical Medical College of Lanzhou University; c Department of Orthopedics, Lanzhou University Second Hospital, Lanzhou, China.

**Keywords:** COVID-19, gastrointestinal disease, liver disease, meta-analysis, SARS-CoV-2

## Abstract

**Background:**

Digestive diseases have been often reported in COVID-19 patients, but whether COVID-19 patients with existing digestive comorbidities are at an increased risk of serious disease and death remains unclear. This study aims to evaluate the association between digestive diseases and COVID-19 severity and mortality.

**Methods:**

PubMed, Embase.com, the Cochrane Central Register of Controlled Trials, Web of Science, China National Knowledge Infrastructure, Wanfang, and SinoMed will be searched to identify relevant studies up to October 1, 2020. We will use the Newcastle-Ottawa quality assessment scale to assess the quality of included studies. We will use Stata to perform pairwise meta-analyses using the random-effects model with the inverse variance method to estimate the association between digestive diseases and the mortality and severity of COVID-19. Subgroup analyses and sensitivity analyses will be conducted to investigate the sources of heterogeneity. We will create a “Summary of findings" table presenting our primary and secondary outcomes using the GRADEpro Guideline Development Tool software.

**Results:**

The results of this study will be published in a peer-reviewed journal.

**Conclusions:**

This study will comprehensively evaluate the association between digestive diseases and the severity and mortality of patients with COVID-19. The results of this study will provide high-quality evidence to support clinical practice and guidelines development.

## 1 Introduction

Coronavirus disease 2019 (COVID-19), first appeared in December 2019, is an acute pneumonia caused by severe acute respiratory syndrome coronavirus 2 (SARS-CoV-2) infection.^[1–3]^ It spreads rapidly to several countries and has become an epidemic.^[4]^ As of September 21, 2020, >30.6 million COVID-19 cases and 950,000 deaths have been reported globally.^[5]^ Furthermore, from September 14 to 20, there were nearly 2 million new cases of COVID-19, the highest number of cases reported in a single week since the beginning of the epidemic.^[5]^

In addition to respiratory symptoms, gastrointestinal comorbidities have been often reported in patients with COVID-19.^[6]^ A previous meta-analysis suggested that the prevalence of chronic liver diseases was significantly associated with more severe COVID-19 infection (odds ratio [OR] = 1.48, 95% confidence interval [CI]: 1.17–1.87; *P* = .001) and overall mortality (OR = 1.78, 95% CI: 1.09–2.93, *P* = .02).^[7]^ Another meta-analysis revealed that patients with severe COVID-19 disease had higher rates of liver injury (OR = 2.20, 95% CI: 1.60–3.02; *P* < .00001) compared nonsevere patients, and patients with gastrointestinal involvement had a higher prevalence of complication (OR = 2.51, 95% CI: 1.62–3.89; *P* < .0001).^[8]^ However, previous meta-analyses evaluated the association between gastrointestinal diseases and COVID-19 severity and mortality based on limited sample sizes. Furthermore, some digestive comorbidities, such as constipation, cholelithiasis, and irritable bowel disease, that may affect the severity and mortality of COVID-19 patients have not been studied in previous meta-analyses. And almost all the data in the previous meta-analyses came from China, which may affect the dissemination of the evidence. Therefore, it is necessary to conduct a meta-analysis to comprehensively evaluate whether COVID-19 patients with digestive comorbidities are at an increased risk of serious disease and death.

## 2 Methods

This review protocol has been registered in the International Prospective Register of Systematic Reviews (PROSPERO, CRD42020215453). This systematic review will be performed in accordance with the Preferred Reporting Items for Systematic Reviews and Meta-Analyses (PRISMA) statement.^[9]^ We report this protocol following the preferred reporting items for systematic review and meta-analysis protocols (PRISMA-P) checklist.^[10]^

### 2.1 Inclusion and exclusion criteria

We will include cohort studies focused on COVID-19 patients that compared the prevalence of at least 1 specific digestive comorbidity among infected patients with severe and non-severe disease or between non-survivors and survivors. The COVID-19 definition of severe respiratory infection from the World Health Organization will be used: fever or suspected respiratory infection, plus 1 of the following: respiratory rate >30 breaths/min, severe respiratory distress, or peripheral oxygen saturation (SpO_2_) ≤93% on room air.^[11,12]^ The studies should be published in English or Chinese and the sample size should be >10 patients.

We will exclude the following studies: studies included both confirmed and suspected cases; studies only provided the overall prevalence of digestive diseases without a detailed digestive disease; the prevalence of digestive diseases is provided, but no comparison is made between populations (eg, severe versus nonsevere patients); animal studies, letters, comments, abstracts, editorials, and reviews.

### 2.2 Outcomes

The primary outcome is the association between each digestive disease and COVID-19 severity. The secondary outcome is the association between each digestive disease and COVID-19 mortality.

### 2.3 Search strategy

PubMed, Embase.com, the Cochrane Central Register of Controlled Trials (CENTRAL) and Web of Science will be searched to identify relevant studies up to October 1, 2020. We will also search Chinese databases: China National Knowledge Infrastructure (CNKI), Wanfang, and SinoMed. The keywords will be used include “coronavirus disease-19,” “coronavirus disease 2019,” “2019 novel coronavirus,” “COVID-19,” “severe acute respiratory syndrome coronavirus 2,” “SARS-CoV-2,” “2019-nCoV,” “nCoV-2019,” “novel coronavirus,” “new coronavirus,” “novel coronavirus pneumonia,” “liver disease,” “hepatic disease,” “digestive disease,” “gastrointestinal disease,” “hepatitis,” “cirrhosis,” “gastritis,” “cholelithiasis,” “clinical characteristic,” “clinical feature,” “risk factor,” “prognosis,” “comorbidity.” Reference lists of relevant systematic reviews and eligible studies will be also manually searched to identify additional eligible studies. The search strategy of Embase.com is presented in Table 1.

**Table 1:** Search strategy of Embase.com.

#1 ’covid 19’/exp OR 'severe acute respiratory syndrome coronavirus 2’/exp
#2 ’COVID-19’:ab,ti OR ’COVID 19’:ab,ti OR ’2019-nCov’:ab,ti OR 'SARS-CoV-2’:ab,ti OR ’2019 novel coronavirus’:ab,ti OR ’coronavirus disease 2019’:ab,ti OR ’coronavirus disease-19’:ab,ti OR 'severe acute respiratory syndrome coronavirus 2’:ab,ti OR ’new coronavirus’:ab,ti OR ’nCoV-2019’:ab,ti OR ’novel corona virus’:ab,ti OR ’novel coronavirus pneumonia’:ab,ti
#3 #1 OR #2
#4 clinical characteristics:ab,ti OR clinical characteristic:ab,ti OR clinical feature:ab,ti OR clinical features:ab,ti OR risk factors:ab,ti OR risk factor:ab,ti OR prognosis:ab,ti OR comorbidit^∗^:ab,ti
#5 liver disease^∗^:ab,ti OR hepatic disease^∗^:ab,ti OR gastrointestinal disease^∗^:ab,ti OR digestive disease^∗^:ab,ti OR hepatitis:ab,ti OR cirrhosis:ab,ti OR irritable bowel disease^∗^:ab,ti OR fatty liver:ab,ti OR gastritis:ab,ti OR gastrointestinal haemorrhage:ab,ti OR gastrointestinal hemorrhage:ab,ti OR cholelithiasis:ab,ti
#6 #4 OR #5
#7 #3 AND #6

### 2.4 Study selection

Two review authors will independently screen titles and abstracts of all studies identified from the literature search. For records that cannot be determined by titles and abstracts, we will refer to their full-text reports. Then, 2 review authors will independently evaluate the full-text articles according to the inclusion criteria. If the 2 review authors are unable to reach a consensus, we will consult a third review author to reach a final decision. The procedure of study identification for inclusion and exclusion will be documented.

### 2.5 Data extraction

We will use a data collection form to extract study characteristics and outcome data, which we will test on a random of 5 studies in the review. One reviewer will abstract data from each included study and a second reviewer will check the data. We will extract the following study information.

- General information: first author, country of the first author, journal name, year of publication, publication language.
- Study characteristics: study setting, recruitment time frame, source of participants, inclusion/exclusion criteria, length of follow-up.
- Participant characteristics: age, sex, ethnicity, sample size, participants lost to follow-up.
- Outcomes: prevalence of digestive comorbidities, including liver disease, fatty liver disease, cirrhosis, hepatitis B, hepatitis C, cholelithiasis, irritable bowel disease, and other digestive diseases; number of severe cases, non-severe cases, non-survivors, and survivors.

### 2.6 Risk of bias

Two review authors will independently assess the risk of bias for each study using the Newcastle-Ottawa quality assessment scale (NOS).^[7]^ Any disagreements will be resolved by discussion, or by involving a third assessor. We will consider studies with <5 stars as low quality, 5 to 7 stars as moderate quality, and >7 stars as high quality. The results of the risk of bias assessment will be displayed in a table.

### 2.7 Statistical analysis

#### 2.7.1 Data synthesis

We will use Stata (13.0; Stata Corporation, College Station, TX) for pooling data and for statistical analysis. Pairwise meta-analyses will be conducted to calculate the OR and 95% CI to estimate the association between digestive diseases and the mortality and severity of COVID-19. We will use the random-effects model with the inverse variance method. The statistical level of significance will be set at *P* < .05.

#### 2.7.2 Assessment of heterogeneity

We will carry out tests to explore the heterogeneity between studies using the χ^2^ test. The *I*^2^ statistic will be used to quantify the statistical heterogeneity. We will consider *I*^2^ values of <25%, 26%–50%, and >50% as low, moderate, and high degrees of heterogeneity, respectively.^[13]^

#### 2.7.3 Subgroup analyses

We plan to perform subgroup analyses between different countries, sexes, or ages to explore the potential sources of heterogeneity.

#### 2.7.4 Sensitivity analysis

We will perform sensitivity analyses by excluding studies published in Chinese or lower quality studies to assess the robustness of our conclusions.

#### 2.7.5 Publication bias

The publication bias will be explored using the funnel plot and Egger test for outcomes with studies no <10.

### 2.8 Certainty of evidence

We will create a “Summary of findings" table presenting our primary and secondary outcomes using the GRADEpro Guideline Development Tool (GDT) software.^[14]^ We will use the five Grading of Recommendations Assessment, Development, and Evaluation (GRADE) considerations (risk of bias, inconsistency, imprecision, indirectness, and publication bias) to assess the quality of the body of evidence for each meta-analysis.^[15–17]^ We will rate the quality of evidence as high, moderate, low, or very low, and will justify decisions to downgrade or upgrade the quality of the evidence using footnotes where necessary.

## 3 Results

### 3.1 Screening results

A total of 5862 records were identified through preliminary literature searches. We piloted the study selection process and initially included 10 studies for a pilot analysis. Two reviewers will perform the titles, abstracts, and full-texts screening, and we will present the screening process in a PRISMA flow plot.

### 3.2 General characteristics and quality of studies

The main characteristics and quality of the included studies are shown in Table 2. All included studies were published in English and all were from China. The sample size of each study ranged from 102 to 339. The included studies were rated 5 to 8 stars according to the NOS scale.

**Table 2:** Characteristics of included studies.

First author	Year	Country	Language	Recruitment time frame	Sample	Male, N (%)	Age^∗^	NOS
Cao et al^[18]^	2020	China	English	2020.1.3–2020.2.1	102	53 (52.0)	54 (37–67)	6
Chen et al^[19]^	2020	China	English	2020.1.1–2020.2.10	203	108 (53.2)	54 (41–68)	6
Hu L^[23]^	2020	China	English	2020.1.8–2020.2.20	323	166 (51.4)	61 (23–91)	8
Wan SX^[24]^	2020	China	English	2020.1.23–2020.2.8	135	72 (53.3)	47 (36–55)	7
Wang et al(a)^[20]^	2020	China	English	−2020.2.10	107	57 (53.3)	51 (36–65)	8
Wang et al (b)^[25]^	2020	China	English	2020.1.1–2020.1.28	138	75 (54.3)	56 (42–68)	8
Wang L^[21]^	2020	China	English	2020.1.1–2020.2.6	339	168 (49.6)	69 (65–76)	5
Wu J^[26]^	2020	China	English	2020.1.20–2020.2.20	280	151 (53.9)	43.1 (19.0)	7
Yan et al^[22]^	2020	China	English	2020.1.10–2020.2.24	193	114 (59.1)	64 (49–73)	7
Zhang et al^[27]^	2020	China	English	2020.1.2–2020.2.10	221	108 (48.9)	55 (39–66.5)	7

### 3.3 Association between chronic liver disease and mortality of COVID-19

**Figure 1:**
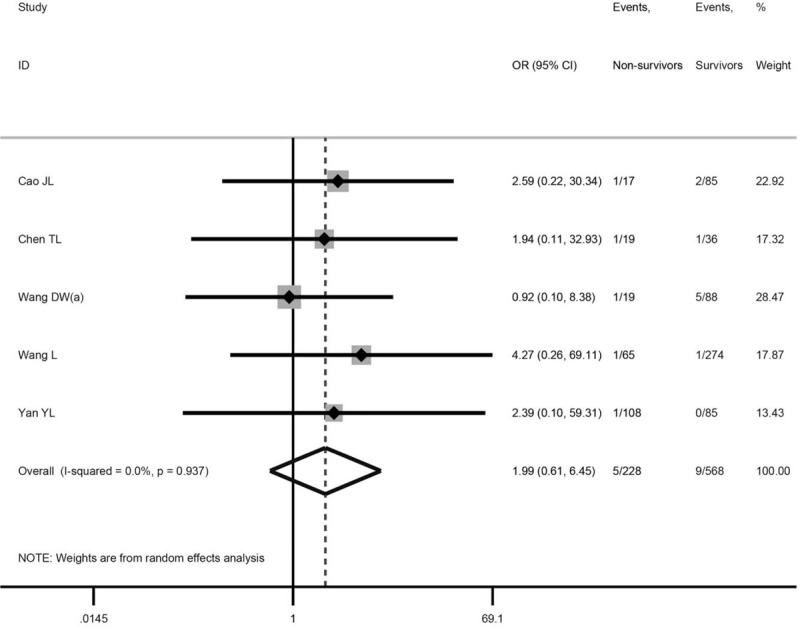
Association between chronic liver disease COVID-19 mortality.

We found no significant difference between non-survivors and survivors in the prevalence of chronic liver disease (5 studies,^[18–22]^ 796 patients; OR = 1.99, 95% CI: 0.61–6.45, *P* = .253; *I*^2^ = 0.0%). Some of the results are shown in Figure 1.

### 3.4 Association between chronic liver disease and the severity of COVID-19

**Figure 2:**
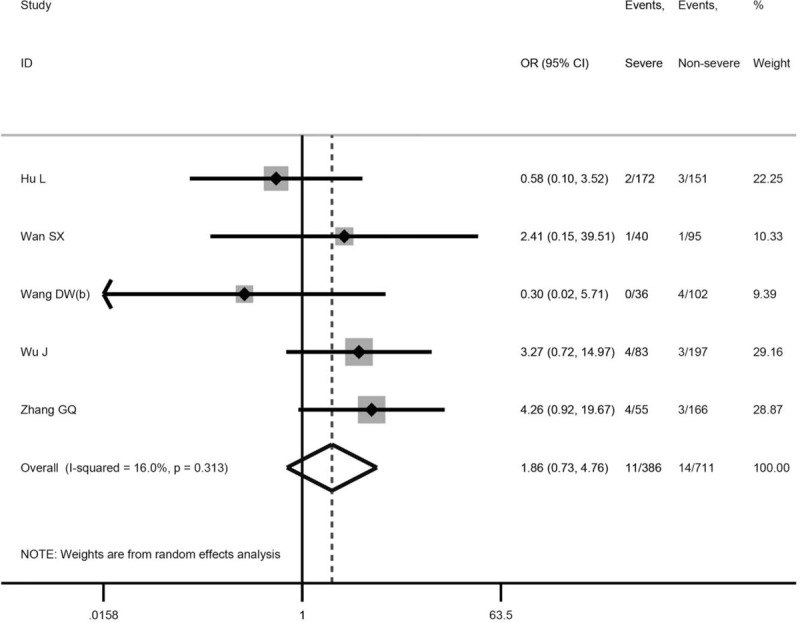
Association between chronic liver disease COVID-19 severity.

Compared with patients without severe disease, those with severe disease were more likely to have chronic liver disease (5 studies,^[23–27]^ 1097 patients; OR = 1.86, 95% CI: 0.73– 4.76, *P* = .195; *I*^2^ = 16.0%, Fig. 2).

## 4 Discussion

Empirical evidence demonstrated that liver damage is an important risk factor for severe outcome and death in SARS and MERS.^[28,29]^ Previous studies indicated SARS-CoV-2 can enter the host cells by binding to the angiotensin-converting enzyme 2 (ACE2) receptor.^[30]^ Thus, it is urgent to investigate whether existing liver diseases and other digestive comorbidities are associated with COVID-19 prognosis. What is more, having a reliable estimate of the association between digestive diseases and COVID-19 severity and mortality is also crucial to ensure whether special protective measures and treatment options should be provided for patients with digestive comorbidities during the COVID-19 pandemic.^[31]^ We believe the results of our study will provide high-quality evidence to support clinical practice.

## Author contributions

**Conceptualization:** Jinjuan Li, Shunan Zhang, Rongna Lian, Ruinian Zhang.

**Funding acquisition:** Peng Cheng.

**Investigation:** Shunan Zhang, Jianjun Wu.

**Methodology:** Jianjun Wu, Peng Cheng.

**Project administration:** Jianjun Wu.

**Resources:** Jinjuan Li, Jia Yue.

**Validation:** Jianjun Wu.

**Visualization:** Jia Yue, Rongna Lian, Ruinian Zhang.

**Writing – original draft:** Jinjuan Li, Jianjun Wu.

**Writing – review & editing:** Jinjuan Li, Jianjun Wu.
